# Charge carrier thermalization in organic diodes

**DOI:** 10.1038/srep19794

**Published:** 2016-01-21

**Authors:** N. J. van der Kaap, L. J. A. Koster

**Affiliations:** 1 Zernike Institute for Advanced Materials, University of Groningen, Nijenborgh 4, 9747 AG, Groningen, The Netherlands

## Abstract

Charge carrier mobilities of organic semiconductors are often characterized using steady-state measurements of space charge limited diodes. These measurements assume that charge carriers are in a steady-state equilibrium. In reality, however, energetically hot carriers are introduces by photo-excitation and injection into highly energetic sites from the electrodes. These carriers perturb the equilibrium density of occupied states, and therefore change the overall charge transport properties. In this paper, we look into the effect of energetically hot carriers on the charge transport in organic semiconductors using steady state kinetic Monte Carlo simulations. For injected hot carriers in a typical organic semiconductor, rapid energetic relaxation occurs in the order of tens of nanoseconds, which is much faster than the typical transit time of a charge carrier throught the device. Furthermore, we investigate the impact of photo-generated carriers on the steady-state mobility. For a typical organic voltaic material, an increase in mobility of a factor of 1.1 is found. Therefore, we conclude that the impact of energetically hot carriers on normal device operation is limited.

Organic semiconducting conjugated polymers and small molecules can be used for fabricating low-cost electronic devices such as solar cells, light emitting diodes and transistors^1^,^2^,^3^. The short delocalization distances and the high amounts of imperfections prevent band transport. Instead, charge transport is based on phonon mediated hopping of carriers between localized segments of the conjugated molecule^4^. The energetic distribution of the localized segments is characteristic for the material under investigation, and is often translated into a gaussian density of states (DOS) with a standard deviation of *σ*^5^.

In thermal equilibrium and for vanishing charge densities, charge carriers will on average reside at the equilibrium level of 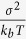 below the center of the DOS, where *k*_*b*_ is the Bolzmann constant and *T* is the temperature^5^. For finite charge concentrations and no electric field, the particles will arrange according to the Fermi-Dirac distribution. In the presence of an electric field, this distribution is perturbed slightly^6^. Although some charges will reside in high energetic states, this concentration is constant and relatively low. Transient techniques like time-of-flight experiments have shown that charge transport is prone to dispersion: carriers in highly energetic states move faster than carriers that are located at low energetic sites^7^. These experiments are supported by kinetic Monte Carlo (KMC) simulations of particles that hop through a cubic lattice^8^. Nevertheless, charge transport in real materials is often characterized by steady-state mobilities. These mobilities include dispersion effects as long as charges are in an energetic steady-state equilibrium, but fail once carriers do not obey the equilibrium distribution. Experimentally, these mobilities can be obtained by analysis of space charge limited currents (SCLC) in diodes consisting of an organic layer that is sandwiched between two electrodes^9^. The current density *J* in this type of device is limited by an accumulation of space charge, and is given by^10^:


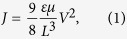


where *μ* is the mobility, *L* the device thickness, and *V* the applied voltage. Because *J* is proportional to *μ*, the mobility can be determined from the current density. Experiments also replicate the accompanying thickness scaling of the mobility, which supports the validity of using steady state mobilities in organic semiconductors^11^. The mobilities found from SCLC analysis have also been applied successfully to organic photovoltaics (OPV). Mihailetchi *et al*. showed a clear correlation between the mobility and the power conversion efficiency of solar cells^12^; Proctor *et al*. found that high fill factors can only be achieved for steady state electron and hole mobilities exceeding 10^−4^ cm^2^V^−1^s^−1^^13^; and Barthesaghi *et al*. showed that the fill factor depends directly on the steady state mobilities^14^. Moreover, drift-diffusion simulations accurately describe the charge transport characteristics in organic light emitting diodes, single carrier diodes and transistors^15^,^16^,^17^. This indicates that steady-state mobilities are definitely related to the operation of these devices.

Recently, it was proposed that charge extraction in organic photovoltaic devices is governed by thermalization of photo-generated highly energetic (hot) carriers^18^. After dissociation, the steady-state distribution is perturbed since an increased amount of charge resides in high energetic states, allowing fast cascaded transport of hot carriers towards the extracting electrodes (Fig. 1a). This process enhences the overall charge transport. Immidiately after dissociation, the mobility is large, but it decays towards a steady-state value once the energetic steady-state distribution is restored. These results agree with the work of Bässler on energetic relaxation in time of flight measurements of disordered semiconductors, which predicts that relaxation times scale exponentially with the level of disorder^7^. Because the energetic relaxation times are much longer than the average transit time of photo-generated carriers to the respective electrode, these carriers will never reach the transport regime that is described by the equilibrium mobility^18^. This raises the question why the analysis of SCLC data still appears to work, what is really measured in such an experiment, and what the influence of thermal relaxation on charge transport is in sandwiched devices.

To this end, we perform KMC calculations on charge transport in sandwiched single carrier diodes, where the work function of the metallic electrodes aligns with the center of the DOS. Although this type of device does not contain photo-generated carriers, the energetic steady-state distribution of charges is perturbed by introducing high-energetic charges that are injected from the electrodes. Figure 1b shows the energy diagram of the interface between a disordered material and a metal that aligns with the center of the density of states. Injection rates into all sites below the center of the DOS can all considered to be high. Because the number of available states just below the center of the density of states is much larger than the number of states around the equilibrium level, most injected carriers will end up here, and are thus considered as hot. Therefore, this approach allows relaxation effects to be investigated under operational conditions. Furthermore, hot carrier injection is artificially increased in the KMC simulation by enhancing the injection rates into high energetic locations. This exaggerates the effect of hot carrier relaxation of injected charges. For both situations, we determine the relation between initial energy level of carriers after injection, and the transit time of charges through the device, as well as the energetic relaxation length of individual particles.

We also study the impact of photo-generated carriers on bulk transport, since these carriers introduce a perturbation of the energetic steady-state distribution as well. This is done by simulating a bulk region of the the material, where relaxed charge carriers are periodically re-excitated to a high energy level. After discussing the impact of injected hot carriers, we also study the influence of photo-generated hot carriers on the steady-state charge carrier mobility in the bulk region of the material. Because the steady-state charge carrier mobility depends on the carrier concentration, the carrier densities need to be fixed. Bimolecular recombination and charge injection and extraction violate this property in real devices. Therefore, a fully periodic KMC simulation volume and only one type of carrier are used. We compare the obtained mobilities with the steady-state mobilities in thermal equilibrium, and determine the origin of the difference.

## Methods

One dimensional drift-diffusion simulations are a computationally cheap method for determining the charge transport characteristics in (organic) semiconducting devices. The simulations are based on numerically solving the coupled 1D Poisson and continuity equations^19^. The equations are discretized for use on a 1D spatial grid, where each node has a certain electric potential *φ*, and quasi Fermi level *η*. Because the simulation uses a generalized version of the Einstein relation for a gaussian density of states, an alternative method is applied to obtain results from the current relation.

The current relation, which returns the current density *J* between two nodes *j* and *j* + 1, given *η*_*j*_, *η*_*j*+1_, *φ*_*j*_ and *φ*_*j*+1_, is needed for solving the continuity equation. It is possible to directly substitute the current relation in the continuity equation, but solving the resulting system is mathematically challenging. Instead, we use a Scharfetter-Gummel approach that decouples the Poisson equation from the continuity equations^20^. The values for the current density between all grid nodes are obtained by solving


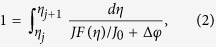


where *F*(*η*) is the Fermi-Dirac integral and *J*_0_ is a constant given by 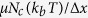
21. Here, *μ* is the charge carrier mobility, *N*_*c*_ is the density of states, *k*_*b*_ is the Boltzmann constant, *T* is the temperature and Δ*x* is the spacing between the grid points.

After initializing the simulation, each iteration starts by solving the Poisson equation. Next, Eq. 2 is solved and the resulting values are used for solving the continuity equation. This process is repeated until the current between all grid nodes has converged. The Poisson equation and the continuity equation are both linear first order equations, and can be expressed in terms of tridiagonal matrices. This allows for a fast solution using gaussian elimination. The drift-diffusion simulation uses Fermi-Dirac statistics including a gaussian density of states and the generalized Einstein relation^22^. The dependence of mobility on electric field, disorder and disorder is given by an interpolation table that contains results from periodic KMC simulations including short-range Coulomb interactions.

KMC simulations calculate the charge dynamics of individual particles, rather than using macroscopic quantities like carrier density and mobility^7^. The simulation volume consists of a 3D cubic lattice, where each node represents a hopping site^7^. The lattice constant *a* is set to the average hop distance in organic semiconductors, and each node is given a gaussian distributed energy level with a standard deviation of *σ*eV. This type of simulation has helped to find many features of charge transport in disordered materials^5^,^7^,^23^. In the *x*- and *y*-directions, periodic boundary conditions apply, and 256 × 256 hopping sites are used. The number of sites in the *z*-direction depends on the device thickness, and metallic electrodes are assumed next to the bottom and top sites of the grid. Two hoprate expressions are used during the simulations that determine the hoprate *ν*_*ij*_ as a function of the energy difference Δ*E*_*ij*_ between sites *i* and *j*: Miller-Abrahams rates


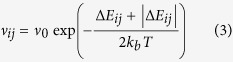


and Marcus rates^24^,^25^.


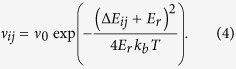


Here, *ν*_0_ is the attempt to jump frequency and *E*_*r*_ is the reorganization energy. Miller-Abrahams rates were developed for describing hopping transport through electron traps in inorganic semiconductors and are often used because of their simplicity. Marcus rates include an energetic barrier between the initial and final hop states, which originates from energetic relaxation due to the polarization of the surroundings of the charge. Marcus rates are more realistic, but introduce an additional parameter to the simulation.

For the calculations of the short range Coulomb interactions, a cylindrically shaped cutoff region is used, with a height that equals the device thickness, and a radius of 16 lattice spacings in the *xy*-plane. For a hop distance of 1.5 nm, this results in a radius of 24 nm, and is reasonably large^26^. For each charge within the cutoff radius, three image charges are taken into account: the first image on each side of the electrodes, and the second image on the side of the electrode that is closest to the location where the interaction is calculated. This guarantees that the electric field is always perpendicular to the closest contact, and that the potential due to close range interactions is zero at that contact.

The electrodes are treated as ideal metal contacts with a fixed work function. Injection from the electrodes into the closest hopsites of the semiconductor is done in a similar way as normal charge hopping, where the work function of the metallic electrode is used as a site energy. The same is done for charge extraction. In order to increase the impact of hot carrier injection on charge transport, the injection rates into highly energetic sites is artificially increased for some of the calculations as indicated.

For the calculations on the impact of photo-generated hot carriers, a KMC simulation with full periodic boundary conditions is used. The dimensions of this simulation are set to 256 × 256 × 256 nodes, and a spherical cutoff region with a radius of 16 lattice spacings is used for the electrostatic interactions. After the initial placement of a fixed number of charges, the simulation is started for a specified generation rate *G* of photo-generated charge carriers. Every 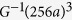 seconds, the carrier that covered the most distance in the electric field direct is replaced by a randomly introduced charge. This mimics the extraction of the carrier that was most likely to leave the device, and is necessary in order to keep the overall charge concentration constant. The simulation is run until the mobility converges to an equilibrium value. After equilibrium has been reached, the energy levels of all new hot carriers are traced to obtain insight in the relaxation dynamics.

In order to acquire a realistic set of simulation parameters, a hole-only diode was fabricated and measured. First, a 40 nm thick layer of PEDOT:PSS, a water-based suspension of poly(3,4-ethylenedioxythiophene) stabilized with poly(4-styrenesulphonic acid), was spincast on a glass substrate patterned with indium tin oxide. On top of the PEDOT:PSS, a 100 nm thick layer of poly(3-hexylthiophene-2,5-diyl) (P3HT) was deposited by spincoating from a 15 mg/ml solution in chlorobenzene. The device was finished by thermally evaporating a molybdenum(VI) oxide electrode with a thickness of 10 nm, capped by 100 nm of aluminum. Next, the current-voltage characteristics of the device were measured using a Keithley 2400 sourcemeter.

## Results/Discussion

**Figure 2:**
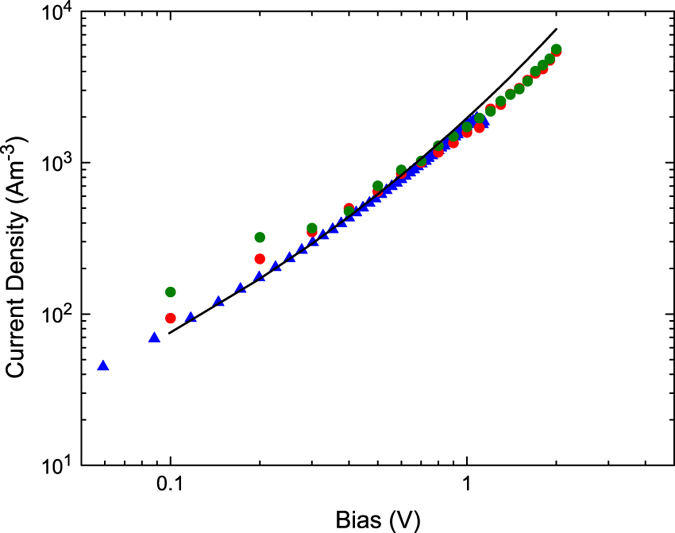
Fits to experimental current voltage characteristics of a P3HT hole-only diode (blue triangles). The black line shows the fit using the drift-diffusion simulation. The red circles show the fit using a KMC simulation with Miller-Abrahams hoprates and the green circles show the KMC fit using Marcus hoprates.

The operation of the KMC simulation is confirmed by modeling the charge transport through the P3HT hole-only diode without injection barriers and with a thickness of 100 nm. The experimental data are reproduced by both the 1D drift-diffusion simulation and the KMC simulations. In order to do so, a device with an energetic disorder *σ* of 0.10 eV and ohmic injection is modeled at a temperature of 295 K. The hopping distance equals 1.5 nm and *ν*_0_ is set to 9 × 10^10^ s^−1^ and 2.7 × 10^11^ s^−1^ for Miller-Abrahams rates and Marcus rates respectively. For the simulations including Marcus rates, a reorganization energy of 0.25 eV is used, which is a common value in OPV^27^. The resulting current-voltage characteristics are shown in Fig. 2. The simulation results give a unique fit to the experimental results, and the adopted parameters are physically realistic. During the remaining calculations, we will therefore use the same parameter set.

**Figure 3:**
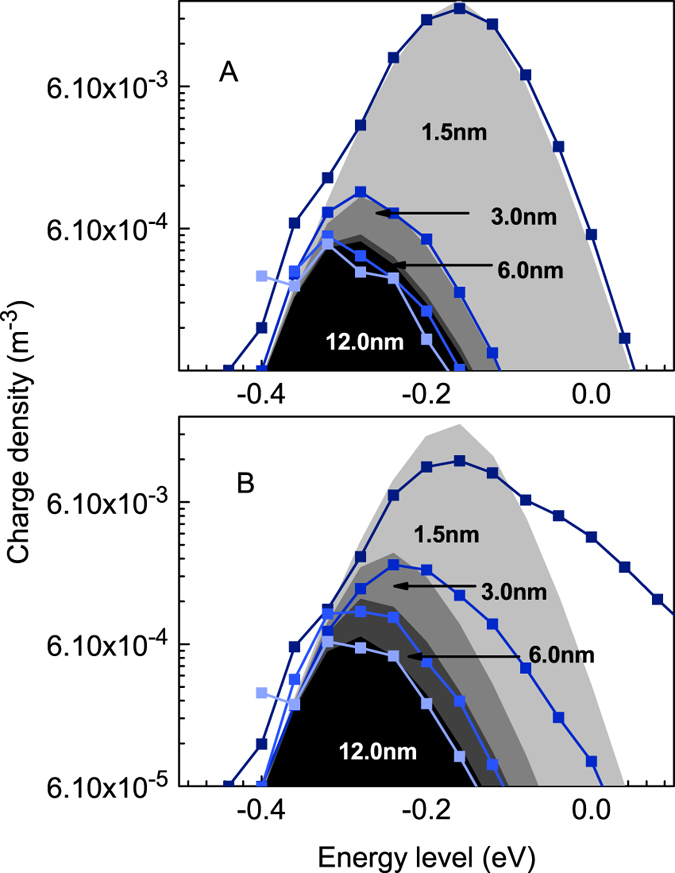
Energetic distribution of charges at the indicated distance from the injecting electrode, for regular injection (A) and artificially increased injection of hot carriers (**B**). The lines show the extracted distribution from the simulation, while the filled curves are predicted theoretically by the Fermi-Dirac distribution. In figure (**B**), it can clearly be seen that the energetic distribution of charge carriers is perturbed. However, this deviation from Fermi-Dirac statistics has already diminished after 6 nm into the device.

First, calculations on the injection of hot carriers are discussed. The energetic distribution of carriers that are close to the injection electrode is calculated for both the normal and enhanced injection approaches. Figure 3 shows the results of the calculations that were performed using Miller-Abrahams hopping rates. The symbol lines represent the energetic distribution of charges at the indicated distance from the electrode, whereas the shaded regions correspond to the equilibrium Fermi-Dirac distribution for the same carrier density. For non-vanishing electric fields, charges will distribute according to a an adapted Fermi-Dirac distribution that uses an increased effective temperature^6^. In the work by Cottaar *et al*., an accurate parameterization for this effect was developed^28^. For the relatively small fields that are used in the calculations (0.3*σ*(*qa*)^−1^ Vm^−1^), the parameterization is almost identical to the Fermi-Dirac distribution. This justifies the comparison of the Monte Carlo calculations with the values obtained from the Fermi-Dirac distribution.

For the case of regular injection, all carriers are distributed according to the Fermi-Dirac distribution. This indicates that relaxation occurs very fast and does not influence charge transport. When enhanced hot carrier injection is included, an increased concentration of high energetic carriers is observed close to the injecting electrode. Although the difference with the theoretical distribution is large at first, it vanishes after only a few nanometers in to the device: at a distance of 6 nm from the electrode, all charges are in thermal equilibrium.

Next, the dynamics of hot carriers are investigated. To determine whether these charges traverse the device faster than other charges do, the transit time between injection and extraction of all injected carriers is tracked for an applied bias of 2 V. Analysis of the data shows no correlation between the energy level after injection and the transit time or the number of required hops for reaching the opposite contact. This indicates that hot carriers have the same transit time as ordinary carriers, and that energetic relaxation of injected hot carriers is unlikely to play a major role in SCLC devices.

**Figure 4:**
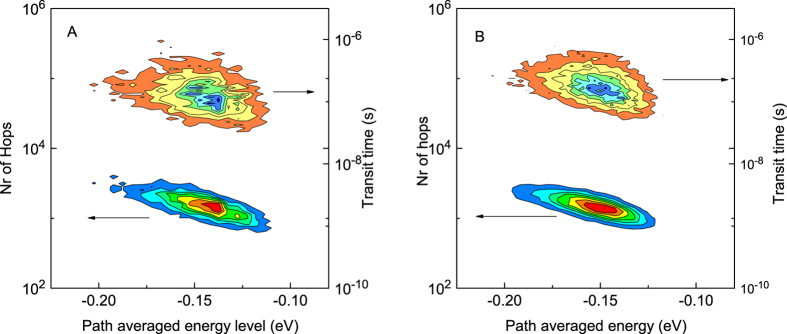
Correlation between the path averaged energy of a particle, the required number of hops, and the required transit time. Calculations were performed for both Miller-Abrahams hopping (**A**) and Marcus hopping (**B**) expressions. Colors indicate the number of events. These plots show that the average energy level of a charge during its transit is important for the transit time and number of hops. This illustrates the dispersive nature of the charge transport in disordered materials. No correlation was found between the energy level after injection and the required number of hops, this is an indication that carrier thermalization is not important for the overall charge transport.

Although no correlation is found for the energy level after injection, a relation exists between the transit time and the path averaged energy level of each carrier. This value is obtained by averaging all energy levels that a particles visited between injection and extraction. Figure 4 contains the plots of the path averaged energy level versus transit time and required number of hops. It shows a reduced number of required hops and a lower transit time for particles with a high averaged path energy. This confirms that high energy levels help charge transport. This can be explained by the larger number of available hop sites with a comparable energy level, and the reduced number of available sites with a low site energy.

**Figure 5:**
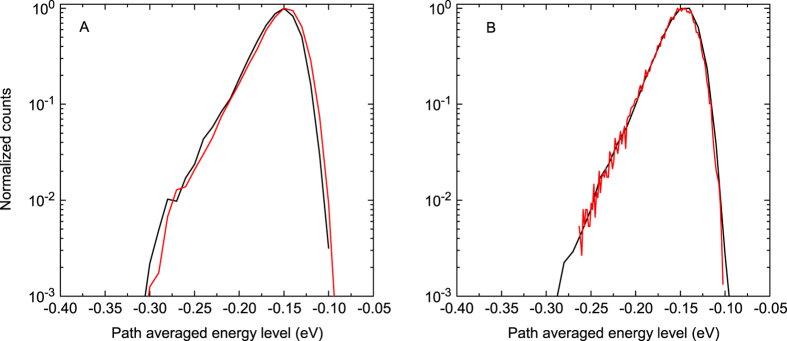
Distribution of the path averaged energy levels for Miller-Abrahams (**A**) and Marcus (**B**) hopping expressions. The black lines correspond to regular injection, while the red lines correspond to artificially increased injection of hot carriers. Highly energetic charges are more mobile, reflecting the effect of dispersion. However, there is no difference between the case with normal injection and increased injection. This indicates that injected hot carriers do not have an increased path averaged level.

A test was performed to verify that no correlation exists between the path averaged energy level and to the energy level after injection. Furthermore, Fig. 5 contains the distribution of path averaged energy levels for all extracted charges, both for the simulations with and without enhanced hot carrier injection. It shows that the averaged path energy is distributed equally, regardless of the presence of enhanced hot carriers. This proves that the path averaged energy level is independent of the energy level after injection.

**Figure 6:**
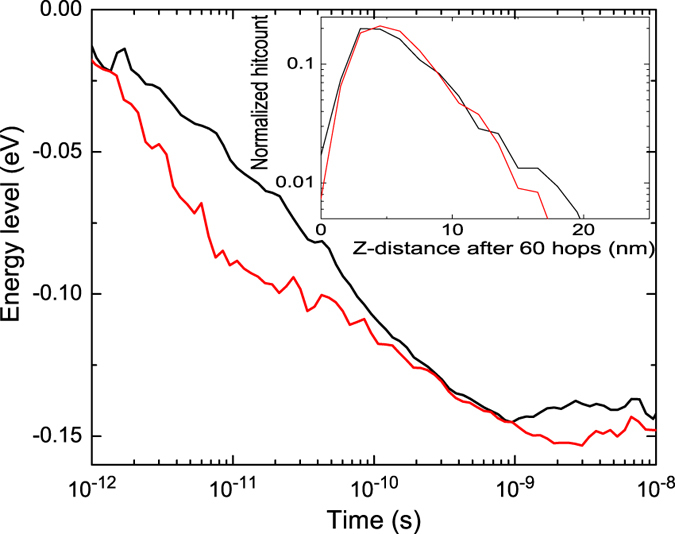
Energetic relaxation of injected hot carriers for Miller-Abrahams (black) and Marcus (red) lines. The relaxation time at 5 ns takes on average 70 hops. Inset: distribution of the z coordinate of injected hot carriers after 60 hop events, using Miller-Abrahams (black line) and Marcus (red line) hopping. This indicates that hot carriers do not travel in a straight line after injection, and do not gain from cascaded transport.

To obtain more insight in the energetic decay of individual charges, the energetic trajectory of individual carriers is followed. The first 60 hops of each hot carrier are followed while recording the energy level and time of occurrence. These data are then averaged over approximately 3,000 hot carriers. The results of these calculations are shown in Fig. 6, both for Miller-Abrahams and Marcus hop rates. Particles loose most of their energy in the first few hops, but show a gradual decrease afterwards. The time required to reach the equilibrium level reaches between 5 and 10 ns, which corresponds to approximately 70 hopping events. This is between one and three orders of magnitude faster than the complete transit time through the device As with the relaxation times in periodic systems, we expect this number to drop for increasing disorder^7^. However, commonly used values for the disorder in P3HT range between 0.071 eV and 0.12 eV^18^,^29^. Therefore, the deviation is expected to be limited. In time delayed collection field (TDCF) experiments, which also include hot carrier effects^30^, the first few nanoseconds cannot be fitted using a drift-diffusion simulation. As this timescale agrees with the relaxation time that we obtained, this effects may be attributed to carrier relaxation. This would also imply that relaxation in fast, and that hot carriers play no role in this type of experiment. The distribution of the z-coordinate of an injected hot carrier after 60 hops provides more evidence for the fast relaxation of these carriers. The inset of Fig. 6 shows that most carriers are still around 5 nm from the injecting electrode. No charges have reached the extracting electrode, although they are almost fully relaxed at this point.

From the calculations so far, it can be concluded that hot carrier injection from the electrodes is not relevant for the charge transport in organic SCL devices. Drift-diffusion calculations including steady-state mobilities from master equation simulations give a very close fit to KMC calculations of the same device^31^. If hot-carrier relaxation was not negligible, the KMC simulation should give much higher values for the current density, because the drift-diffusion simulations do not include carrier dispersion. This outcome was further supported by the fast relaxation of charge carriers near the injecting electrode: within a few nanometers, charges behave according to the Fermi-Dirac distribution again. Furthermore, hot carriers loose most of their energy within the first 70 hops, and traverse 5 hopsites in the electric field direction during this period. Finally, no relations could be found between the energy level after injection and the overall transit time of particles. Instead, a trend was found between the transit time and the path averaged energy level of each particle, were the path averaged energy level is unrelated to the charge carrier being hot or not.

As a next step, we performed calculations on the impact of photo-generated hot carriers on the steady-state charge carrier mobility. The work by Melianas *et al*. includes calculations that were performed on a double carrier device including injection, extraction, generation and recombination of electrons and holes^18^. Moreover, all charge carriers were randomly initialized at the start of the simulation. However, photovoltaic devices usually operate at steady-state conditions under continuous illumination, with the presence of background charges that are in thermal equilibrium. This may change the relaxation dynamics of hot carriers, and lead to different charge transport characteristics. The steady-state charge transport characteristics were calculated by a fully periodic, single carrier KMC simulation. Every 1.8 or 18 ns, the carrier that covered the most distance is reset to a random position, which corresponds to generation rates of 10^28^ m^−3^s^−1^ or 10^27^ m^−3^s^−1^ (approximately the illumination intensity of 1 Sun) for the simulation volume of 5.7 × 10^−20^ m^3^. Because there is no significant difference between the results with Miller-Abrahams and Marcus hoprates in the previous calculations, we only perform the following calculations for Miller-Abrahams hoprates. The dimensionless electric field is set to 0.1, which corresponds to voltage of 0.7 V for a 100 nm device. This is a typical operating voltage for photovoltaic devices.

**Figure 7:**
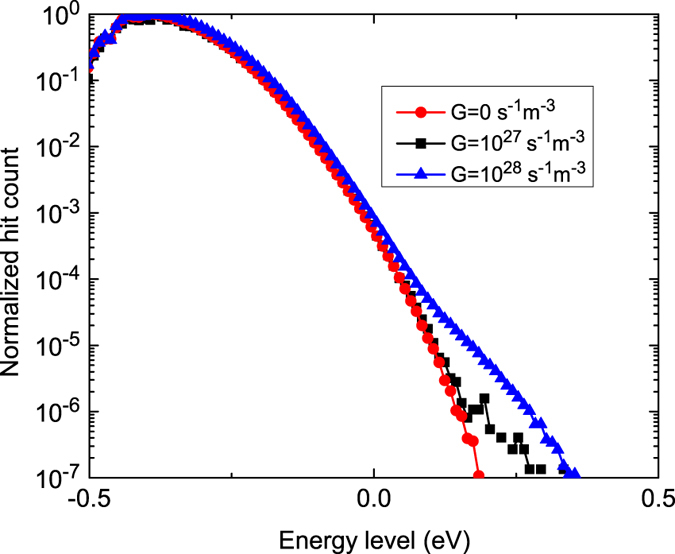
Distribution of charge carriers for different generation rates. For increasing generation rates, the number of highly energetic charges increases. The energetic steady-state distribution is not given by Fermi-Dirac statistics anymore, and the steady-state charge carrier mobility starts to increase.

The simulation is run until the mobility reaches a steady state value. Figure 7 contains the energetic distributions of all charge carriers that are present in the layer, for a typical charge density that is found in OPV (3.1 × 10^−5^*a*^−3^ m^−3^). The periodical introduction of hot charge carriers causes a slight increase in the charge density at high energies. This graph shows the relatively low amount of hot carriers with respect to relaxed carriers. Unlike in the calculations of Melianas *et al*. only one hot carrier is introduced every 1.8 or 18 ns, which reduces the number of hot carriers in the system considerably. Because the number of hot carriers remains equal for different background concentrations, the ratio between hot and relaxed carriers will only decrease for increasing charge concentrations.

**Figure 8:**
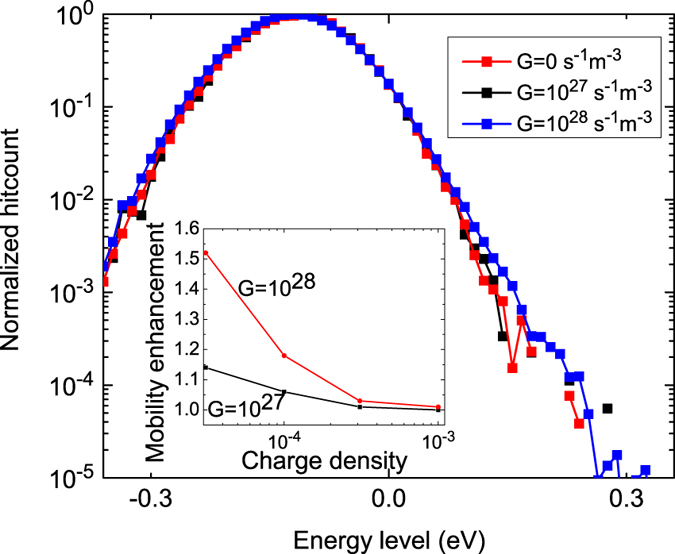
Origin of the current flow through the simulation volume, for different generation rates. It can be seen that charge transport at high energy levels is slightly enhenced when generation is present. Inset: mobility enhancement as a function of charge carrier density, for generation rates of 10^27^ s^−1^m^−3^ and 10^28^ s^−1^m^−3^.

Figure 8 contains the energetic distribution of the mobility with and without generation, for a charge density of 3.1 × 10^−5^*a*^−3^ m^−3^. This graph shows the energy levels of charge carriers that contribute most to the overall transport. The energetic distributions are equal for both methods, which implies that the initial decay through to top of the DOS does not contribute much to the overall current. Moreover, the overall charge transport is mostly determined by charges at an energy level of 0.12 eV below the center of the DOS. Therefore, carrier relaxation to deep energetic sites only forms a small fraction of the overall current. This suggests that carrier relaxation only contributes to the charge transport down to the energy levels where charges still contribute to the mobility.

The inset of Fig. 8 contains the ratio of the steady state mobilities including and excluding the generation mechanism. An increased mobility is found for low densities, while this effect vanishes for higher densities. This occurs do to the reduced ratio of hot carriers for increasing background concentrations. Under the operational conditions of an OPV device, the steady-state mobility is enhanced by a factor 1.1. For a ten times higher generation rate, the difference becomes a factor 1.2. Although this demonstrates the presence of a mobility increase due to photo-generated carriers, this increase is limited to less than one order of magnitude. This also explains why SCLC analyses is still a good method for analyzing OPV devices: the error in experimentally measuring the charge carrier mobility is much smaller than the enhancement due to hot carrier relaxation.

## Conclusion

In conclusion, we investigated the impact of a perturbation of the energetic steady-state distribution of charge carriers on charge transport. This perturbation was either introduced by injection or re-excitation of high energetic (hot) charge carriers. The thermal relaxation of injected hot carriers is found to play a limited role in the operation of single carrier space charge limited devices, for the parameters studied. Hot carriers equilibrate within 5–10 ns after injection. During this time, they only traverse a distance of 5 nm in the electric field direction. Increased numbers are expected for increasing disorder, but the studied parameters are already close to the maximum encountered disorder value of 0.12 eV. Although correlations exist between the transit time, the path averaged energy of charges, and the required number of hops to traverse the device, no relations exist with respect to the initial energy of hot carriers after injection.

Carrier relaxation in bulk material was assessed by a fully periodic KMC simulation, where the location and energy level of charge carriers is randomly reset after fixed intervals. This procedure introduces a deviation from the equilibrium density of states, and increases the number of highly mobile charges. The calculations provide steady-state values for the mobility of devices under continuous illumination. Most of the current flow originates from charge carriers that are located 0.12 eV below the center of the DOS. This implies that carrier relaxation to the deep equilibrium level does not add a large contribution to the overall charge carrier mobility. Under operational conditions, a slightly enhanced steady-state mobility is found that is a factor of 1.1 higher than the steady-state mobility in thermal equilibrium.

Although the presence of hot carrier increases the charge transport characteristics, the enhencement is limited by the relatively low ratio of hot carriers with respect to relaxed charge carriers. The impact of thermal relaxation reduces further when the concentrations of background charges is increased, or when the generation rate is decreased. Finally, these results explain why the steady-state mobilities that are found from space charge limited current analysis provide valuable insight in the device characteristics of OPV devices.

## Additional Information

**How to cite this article**: Kaap, N. J. and Koster, L. J. A. Charge carrier thermalization in organic diodes. *Sci. Rep*. **6**, 19794; doi: 10.1038/srep19794 (2016).

## Figures and Tables

**Figure 1:**
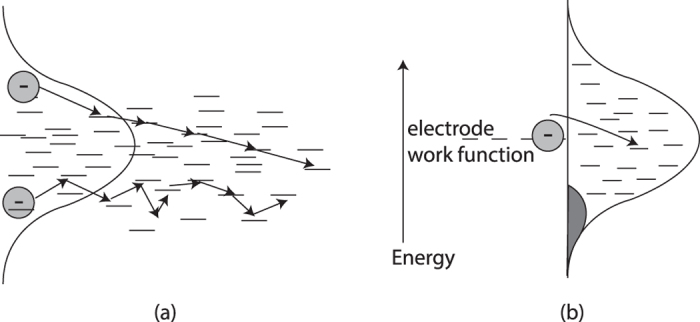
(**a**) Energetic cascade allows fast carrier extraction compared to equilibrium level hopping. (**b**) Injection from metallic electrode into gaussian density of states. The electron hops from the electrode work function to an available site in the semiconductor. The charge is likely to move to a site just below the dashed line: both the hoprates and the number of available states at this level are high.

## References

[b1] Burroughes J. H. . Light-emitting diodes based on conjugated polymers. Nature 347, 539–541 (1990).

[b2] Zhou J. . Solution-processed and high-performance organic solar cells using small molecules with a benzodithiophene unit. J. Am. Chem. Soc. 135, 8484–8487 (2013). 23701031 10.1021/ja403318y

[b3] Sirringhaus H. Device physics of solution-processed organic field-effect transistors. Adv. Mater. 17, 2411–2425 (2005).

[b4] Coropceanu V. . Charge transport in organic semiconductors. Chem. Rev. 107, 926–952 (2007). 17378615 10.1021/cr050140x

[b5] van der Holst J. J. M., van Oost F. W. A., Coehoorn R. & Bobbert P. A. Monte carlo study of charge transport in organic sandwich-type single-carrier devices: Effects of coulomb interactions. Phys. Rev. B 83, 085206 (2011).

[b6] Preezant Y. & Tessler N. Carrier heating in disordered organic semiconductors. Phys. Rev. B 74, 235202 (2006).

[b7] Bässler H. Charge transport in disordered organic photoconductors. Phys. Status Solidi B 175, 15–56 (1993).

[b8] Bässler H. Localized states and electronic transport in single component organic solids with diagonal disorder. Phys. Status Solidi B 107, 9–54 (1981).

[b9] Blom P. W. M., Jong M. J. M. D. & Vleggaar J. J. M. Electron and hole transport in poly (p-phenylene vinylene) devices. Appl. Phys. Lett. 68, 3308–3310 (1996).

[b10] Lampert M. A. Simplified theory of space-charge-limited currents in an insulator with traps. Phys. Rev. 103, 1648 (1956).

[b11] Blom P. W. M., Tanase C., de Leeuw D. M. & Coehoorn R. Thickness scaling of the space-charge-limited current in poly(p-phenylene vinylene). Appl. Phys. Lett. 86, 092105 (2005).

[b12] Mihailetchi V. D., Xie H. X., de Boer B., Koster L. J. A. & Blom P. W. M. Charge transport and photocurrent generation in poly (3-hexylthiophene): methanofullerene bulk-heterojunction solar cells. Adv. Func. Mater. 16, 699–708 (2006).

[b13] Proctor C. M., Love J. A. & Nguyen T. Mobility guidelines for high fill factor solution-processed small molecule solar cells. Adv. Mater. 26, 5957–5961 (2014). 25047697 10.1002/adma.201401725

[b14] Bartesaghi D. . Competition between recombination and extraction of free charges determines the fill factor of organic solar cells. Nat. Commun. 6, 7083 (2015). 25947637 10.1038/ncomms8083 PMC4432638

[b15] Nicolai H. T. . Unification of trap-limited electron transport in semiconducting polymers. Nat. Mater. 11, 882–887 (2012). 22842510 10.1038/nmat3384

[b16] Kuik M. . The effect of ketone defects on the charge transport and charge recombination in polyfluorenes. Adv. Func. Mater. 21, 4502–4509 (2011).

[b17] Brondijk J. J., Torricelli F., Smits E. C. P., Blom P. W. M. & de Leeuw D. M. Gate-bias assisted charge injection in organic field-effect transistors. Org. Electron. 13, 1526–1531 (2012).

[b18] Melianas A. . Dispersion-dominated photocurrent in polymer:fullerene solar cells. Adv. Func. Mater. 24, 4507–4514 (2014).

[b19] Selberherr S. Analysis and Simulation of Semiconductor Devices (Springer, Vienna, 1984).

[b20] Scharfetter D. L. & Gummel H. K. Large-signal analysis of a silicon read diode oscillator. *IEEE Trans*. Electron 16, 64–77 (1969).

[b21] Koprucki T. & Gärtner K. Discretization scheme for drift-diffusion equations with strong diffusion enhancement. Opt. Quant. Electron. 45, 791–796 (2013).

[b22] Wetzelaer G. A. H., Koster L. J. A. & Blom P. W. M. Validity of the einstein relation in disordered organic semiconductors. Phys. Rev. Lett. 107, 066605 (2011). 21902354 10.1103/PhysRevLett.107.066605

[b23] Watkins P. K., Walker A. B. & Verschoor G. L. B. Dynamical monte carlo modelling of organic solar cells: The dependence of internal quantum efficiency on morphology. Nano Lett. 5, 1814–1818 (2005). 16159229 10.1021/nl051098o

[b24] Miller A. & Abrahams E. Impurity conduction at low concentrations. Phys. Rev. 120, 745–755 (1960).

[b25] Marcus R. A. & Sutin N. Electron transfers in chemistry and biology. Biochim. Biophys. Acta 811, 265–322 (1985).

[b26] Casalegno M., Raos G. & Po R. Methodological assessment of kinetic monte carlo simulations of organic photovoltaic devices: The treatment of electrostatic interactions. J. Chem. Phys 132, 094705 (2010). 20210409 10.1063/1.3337909

[b27] Groves C., Kimber R. G. E. & Walker A. B. Simulation of loss mechanisms in organic solar cells: a description of the mesoscopic monte carlo technique and an evaluation of the first reaction method. J. Chem. Phys 133, 144110 (2010). 20949990 10.1063/1.3483603

[b28] Cottaar J., Coehoorn R. & Bobbert P. A. Field-induced detrapping in disordered organic semiconducting host-guest systems. Phys. Rev. B 82, 205203 (2010).

[b29] Ballantyne A. M. . The effect of poly (3-hexylthiophene) molecular weight on charge transport and the performance of polymer:fullerene solar cells. Adv. Funct. Mater. 18, 2373 (2008).

[b30] Albrecht S. . On the field dependence of free charge carrier generation and recombination in blends of pcpdtbt/pc70bm: Influence of solvent additives. J. Phys. Chem. Lett. 3, 640–645 (2012). 26286160 10.1021/jz3000849

[b31] Pasveer W. F. . Unified description of charge-carrier mobilities in disordered semiconducting polymers. Phy. Rev. Lett. 94, 206601 (2005). 10.1103/PhysRevLett.94.206601 16090265

